# Generalized Theory and Decoupled Evaluation Criteria for Unmatched Despreading of Modernized GNSS Signals

**DOI:** 10.3390/s16071128

**Published:** 2016-07-20

**Authors:** Jiayi Zhang, Zheng Yao, Mingquan Lu

**Affiliations:** Department of Electronic Engineering, Tsinghua University, Beijing 100084, China; jiayi-zh15@mails.tsinghua.edu.cn (J.Z.); lumq@tsinghua.edu.cn (M.L.)

**Keywords:** GNSS, signal evaluation, unmatched receiving, anti-interference, decouple

## Abstract

In order to provide better navigation service for a wide range of applications, modernized global navigation satellite systems (GNSS) employs increasingly advanced and complicated techniques in modulation and multiplexing of signals. This trend correspondingly increases the complexity of signal despreading at the receiver when matched receiving is used. Considering the numerous low-end receiver who can hardly afford such receiving complexity, it is feasible to apply some receiving strategies, which uses simplified forms of local despreading signals, which is termed unmatched despreading. However, the mismatch between local signal and received signal causes performance loss in code tracking, which is necessary to be considered in the theoretical evaluation methods of signals. In this context, we generalize the theoretical signal evaluation model for unmatched receiving. Then, a series of evaluation criteria are proposed, which are decoupled from unrelated influencing factors and concentrates on the key factors related to the signal and its receiving, thus better revealing the inherent performance of signals. The proposed evaluation criteria are used to study two GNSS signals, from which constructive guidance are derived for receivers and signal designer.

## 1. Introduction

As global navigation satellite systems (GNSS) have developed and upgraded, new civil signals are designed and employed, in order to support a wide and growing range of navigation services. For the applications including surveying, timing, vehicle navigation and consumer electronics, the level of performance demanded are different. To meet those requirements simultaneously in the modernized GNSS signal for civil use, complicated modulations and multiplexing techniques are employed, represented by Alternative BOC (AltBOC) and multiplexed BOC (MBOC).

These new GNSS signals can be deconstructed with multiple components, thus different level of accuracy can be obtained by processing different number of components. When all of the components are employed to process as an integral, that the local despreading signal is matched with the received signal, the up-bound of the pseudo-ranging accuracy can be obtained. Also, by choosing different number of components or different forms of local despreading signal, a great many strategies are available for the signal receiving. By using these strategies, it is able to promote the positioning accuracy or robustness, such as unambiguous tracking of BOC signal [1] and multipath mitigating techniques [2]. Also, it is possible to reduce the receiver complexity by locally generating a simplified form of the signal, such as the BPSK-like receiving of BOC signal [3,4,5]. Because of these benefits, the various receiving strategies are widely used by receivers in the processing of the MBOC, AltBOC signals.

Theoretical evaluation is an objective and convenient method to acknowledge the performance of a signal. In this context, since the obtained performance is largely associated with the processing techniques implemented by receivers, the theoretical evaluation of signal should also take comprehensive consideration of the receiving strategies. In the aspect of signal design, to provide better performance for a wide range of users, not only the performance under traditional matched receiving, but also the performance under the various pervasive applied receiving strategies, should be considered and optimized, where the theoretical evaluation criteria are treated as the guidance and objective of signal design process. In the aspect of receiving, the theoretical evaluation is a convenient method to compare the performance under various receiving strategies, thus providing useful information for the strategies choosing and configuration for receiver manufactures. Accordingly, the theoretical evaluation of signal performance becomes an essential topic for both the signal design and receiving in GNSS.

In addition, to reveal the properties of signals, the theoretical evaluation criteria should focus on key factors associated with the signal and its processing. A myriad of factors related to the receiving performance can be concluded in the evaluation, including the receiving bandwidth, signal to noise ratio (SNR), interference type and strength, sampling and quantification, etc. However, considering all these factors exaggerate the sophistication of signal evaluating process, making the results coupled with the external factors such as specific working scenarios or receiver implementations, from which it is hard to observe the inherent performance of signal. Therefore, in theoretical evaluation of signals, a concise model decoupled from the unnecessary factors not only increases the objectivity of evaluation, but also facilitates the calculation, which is required by the research in signal design and receiver configuration.

As signal evaluation theory has developed, various routines for choosing the set of related factors in the evaluating model have been proposed. In terms of code tracking evaluation, the initial work comes from communication [6], which is applicable for a BPSK signal under an infinite receiving bandwidth and thermal noise. The paper [7] proposes a model with one equivalent front-end filter and also considers the non-white Gaussian noise and interference. In [8,9], sampling and quantification are added to the evaluation framework, which increase the fidelity and sophistication of the performance evaluation simultaneously. The evaluation model is refined in [10,11], considering the effects of bandlimiting, signal modulation, white Gaussian noise (WGN) and non-white Gaussian interference, which are regarded as key parameters in tracking evaluation. Some of the literature considers the local receiving strategies. Theoretical assessment on tracking and simulation results are provided for Composite BOC (CBOC) in [12]. Tracking jitter under thermal noise in the representation of correlation function is provided in [13]. However, an evaluation method that both supports the various local receiving strategies and decouple from unrelated factors is still lacking in the existing methods.

To solve the abovementioned problems, this paper proposes a theoretical code tracking evaluation method considering various receiving strategies, particularly the mismatch of the code chip waveform of the local despreading signal. The paper also provides a decoupled evaluation criterion termed the “anti-interference rate”. The anti-interference rate, along with equivalent Gabor bandwidth [14], is applied to evaluate modern signals, including MBOC, ACE-BOC, where constructive conclusions are derived for both signal design and receiver configuration. 

The reminder of the paper is organized as following. In the Section “Basic Principles of Code Tracking”, the mathematical model of delay locked loop (DLL) for the code tracking of GNSS receiver is formulated. Then, the “Code Chip Waveform Mismatch” Section gives a theoretical depiction of the unmatched receiving strategies. Based on the model, in the Section “Signal evaluation approach under the local despreading waveform mismatch”, the theoretical performance bound of GNSS signal is obtained considering unmatched receiving. Then in the Section “Decoupled Criteria for GNSS Code Tracking Evaluation”, a series of generalized performance evaluation criteria are derived, which considers the essential factors and decoupled from the less related factors associated with specific implementation and scenarios. Subsequently, in the Sections “The evaluation of unmatched receiving of ACE-BOC” and “The evaluation of BOC-like Receiving of MBOC”, theoretical evaluation is conducted for two modernized GNSS signals, under diverse receiving strategies, from which some useful facts are revealed that will guide the signal design and receiving configuration. Finally, concluding remarks are provided in the Conclusions section.

## 2. Basic Principles of Code Tracking

**Figure 1:**
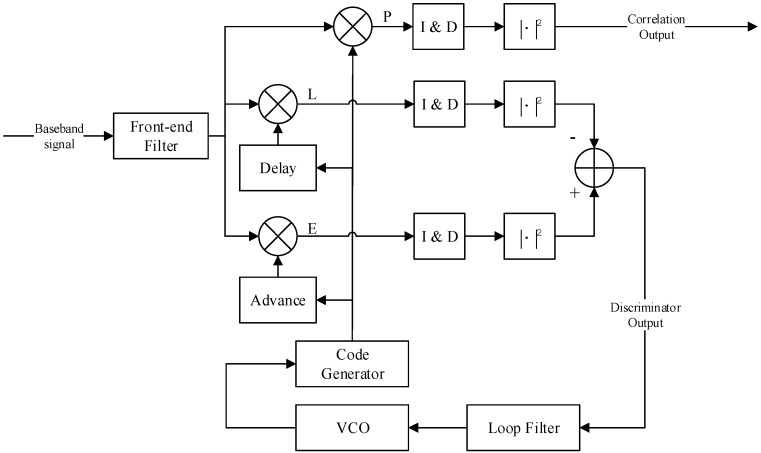
Block Diagram of Non-coherent Code Tracking Loop, where the discriminator model is early minus late power.

Figure 1 shows the diagram of a DLL for pseudo-random noise (PRN) code tracking in the GNSS receiver, where the pseudo range measurement is derived. In this model, a non-coherent early-late loop (NELP) is considered, where an early minus late power discriminator is used.

This work focuses on the performance of code tracking. For the convenience of illustration, assume that the carrier frequency synchronization is perfect. Thus, the input baseband signal of Figure 1 is given as:

(1) $$r(t)=\sqrt{P}e^{j\theta }d(t-\tau_{0})c(t-\tau_{0})+n(t)+\iota (t)$$

where *θ* is the phase difference between local carrier and the received carrier, *P* is the relative signal power, *d*(*t*) is the binary-coded navigation data, *c*(*t*) is the direct sequence spread spectrum (DSSS) waveform assigned to the exact satellite, and $\tau_{0}$ is the true value of time of arrival (TOA), which is regarded to be unchanged within the integration duration *T*.

Also consider the existence of white Gaussian noise (WGN) *n*(*t*) and non-white Gaussian interference *ι*(*t*). The complex envelope of the WGN and interference are represented as a stochastic process, satisfying wide-sense stationary, zero-mean, and circularly symmetry, which are independent of the signal, and statistically uncorrelated with each other [11]. The double-sided spectral density of WGN is denoted as *N*_0_. The power spectral density (PSD) of the interference is denoted as *CιGι*(*f*), with *Gι*(*f*) normalized as $\int_{-\infty }^{+\infty }G_{\iota }(f)=1$. To make the model clear and concise, multipath is not currently shown in Equation (1), while the multipath effects will be discussed in the following sections. 

Further, the spreading waveform *c*(*t*) is defined as:

(2) $$c(t)=\sum_{i=-\infty }^{+\infty }a(i)\varphi (t-iT_{c})$$

where a(*i*) is the PRN spreading sequence, with the value of ±1. φ(t) is the waveform of the current spreading code chip, with the pulse width of *T_c_*.

Then, as shown in Figure 1, the processing of baseband digital signal in the receiver front-end can be equivalent to a transition function *H*(*f*). Here, the assumption of low-pass filter with rectangular amplitude-frequency response is used [11]:

(3) $$H(f)=\{\begin{array}{ll} 1, & |f|<\beta_{r}/2\\ 0, & \mathrm{else} \end{array}$$

where in the double-sideband bandwidth is *β_r_*. After the filter, the received signal is correlated with three branches of the local despreading signals, with the code phase delay of early, prompt and late respectively. To support various receiving strategies, the local despreading code in the model is described in the next section.

## 3. Code Chip Waveform Mismatch

Traditional despreading follows the matched receiving principle that maximizes the SNR at the correlator output [15]. This requires the local despreading signal to be exactly the same as the received signal, depicted as $\hat{c}$(*t*) = *c*(*t*). In GNSS pseudo-range measurements, besides SNR, other indicators including Gabor bandwidth, mean time to lose lock (MTLL) of tracking loop, should also be considered [14,16,17]. Under this background, maximizing SNR and matched receiving is no longer the only guideline to select local despreading signals. In practice, a variety of selections of despreading waveform have been applied in GNSS signal processing, for the purpose of reducing receiver complexity [3,4,5], multipath mitigation [2] and unambiguous tracking of BOC signals [1]. These receiving strategies, which associate with the mismatch of local despreading code chip waveform, are termed as ‘unmatched receiving’ hereafter.

In GNSS, since the PRN spreading sequence is known on both the transmitter side and the receiver side, hereafter we consider a general case where the despreading code chip waveform $\hat{\varphi }(t)$ may be different from that of the received signal, denoted as φ(t). Taking the BOC_sin_(6,1) signal for example, the code chip waveform of the received signal is a bipolar pulse φ(t)$=sign\left\{\sin\left(2\pi f_{sc}t\right)\left[u\left(t\right)-u\left(t-T_{c}\right)\right]\right\}$, where *u*(*t*) is the unit step signal, *f_sc_* is the subcarrier frequency. When a BPSK-like unmatched receiving algorithm is applied, the code chip waveform of local despreading signal is $\hat{\varphi }(t)=u\left(t\right)-u\left(t-T_{c}\right)$ at the central frequency of *f_sc_* or –*f_sc_*, when intermediate frequency is assumed to be zero. Accordingly, BPSK-like receiving is easy to conduct with accuracy loss.

From this aspect, the local despreading signal $\hat{s}\left(t\right)$ is:

(4) $$\hat{s}(t)\;=\;\hat{c}(t)=\sum_{i=-\infty }^{+\infty }a(i)\hat{\varphi }(t-iT_{c})$$

where it is reasonable to ignore the data message for its slow varying characteristics compared with the PRN code chip.

To carry out the despreading of the DSSS signal, correlation is made between the received signal and the local replica. To help represent the characteristics of despreading in the spectrum domain, due to the strict limitation of band for both transmitters and receivers, a Fourier transform is conducted for the cross-correlation result. Within the observation duration (*k*–1)*T_c_* < *t* < *kT_c_*, and for the useful signal term without noise and interference, there is:

(5) $$\frac{1}{T_{c}}\int_{(k-1)T_{c}}^{kT_{c}}E\left\{s(t){\hat{s}}^{*}(t-\tau )\right\}e^{-j2\pi ft}dt\;=\;f_{c\;}\Phi (f){\hat{\Phi }}^{*}(f)≜X(f)$$

where *f_c_* = 1/*T_c_* is the spreading chip rate, X(f) is defined as the cross-spectrum between the received signal and the local signal, Φ(f) is the Fourier transform of the received signal *s*(*t*), and $\hat{\Phi }(f)$is the Fourier transform of the local signal $\hat{s}\left(t\right)$.

As a special case for the matched receiving, wherein the local code chip waveform is identical to that of the received signal, Equation (5) becomes the power spectral density *G_s_*(*f*):

(6) $$C_{s}G_{s}(f)=f_{c}\Phi (f)\Phi^{*}(f)$$

where Φ(f) is normalized to satisfy $\int_{-\infty }^{+\infty }G_{s}(f)=1$, by introducing the average carrier power *C_s_* defined on the infinite bandwidth. Likewise, the local term $\hat{\Phi }(f)$ is commonly normalized to satisfy $\int_{-\infty }^{+\infty }G_{\hat{s}}(f)=1$. It should be noted that, when the average carrier power *C_s_* is derived from the cross-spectrum, that satisfies, $C_{s}X_{s,\hat{s}}\left(f\right)=f_{c}\Phi \left(f\right)\Phi^{*}\left(f\right)$ where both Φ(f) and $\Phi^{*}(f)$ are normalized as mentioned above, so the integration of $X_{s,\hat{s}}\left(f\right)$ on infinite bandwidth is slightly less than 1.

Accordingly, the normalized cross-spectrum $X_{s,\hat{s}}\left(f\right)$characterizes the effect of unmatched receiving, which will be used in the derivation of theoretical evaluation formula in the next section.

## 4. Signal Evaluation Approach under the Local Despreading Waveform Mismatch

### 4.1. Formulation and Statistical Properties of Correlator Output

To evaluate the code tracking accuracy, the mathematical formulation at every module in the diagram of Figure 1 should be obtained, particularly the discriminator output. In the process, correlator output is an intermediate result, which can be used in the evaluation of acquisition and demodulation performance. Different from the existing literature, the evaluation methods provided in this section support unmatched receiving.

Initially, the correlation of the local despreading signal $\hat{s}\left(t\right)$ and received signal *r*(*t*) after integrate and dump (I & D) is provided as:

(7) $$\delta_{i}=\frac{1}{T}\int_{(k-1)T}^{kT}r(t)\;{\hat{s}}^{*}(t-{\hat{\tau }}_{0}+\tau_{i})dt$$

which is derived from the *k*-th integration cycle with the duration *T*, where the subscript i∈{E,P,L} stands for the branches of early, prompt and late, with the code phase delays of $\tau_{E}$ = Δ/2, $\tau_{P}$ = 0 and $\tau_{L}$ = –Δ/2, respectively, where ∆ stands for early-late correlator spacing. $\hat{s}(t-{\hat{\tau }}_{0}+\tau_{i})$ stands for the local signal replica, where ${\hat{\tau }}_{0}$ is the estimated signal delay. Then the error of TOA estimation can be expressed as $\varepsilon =\tau_{0}-{\hat{\tau }}_{0}$.

It should be noted that in traditional matched receiving, the local despreading replica is selected as the conjugate form of the received spreading signal. In this model, however, the local despreading signal $\hat{s}\left(t\right)$ and the received signal *s*(*t*) are allowed to be different in the spreading/despreading code chip waveform, i.e., φ(t) and $\hat{\varphi }(t)$ are allowed to be different, thereby unmatched receiving is supported.

Denote *w*(*t*) as Gaussian noise and interference:

(8) w(t)=n(t)+ι(t)

Define *s*(*t*) as the effective part of signal, then Equation (1) can be rewritten as:

(9) $$r(t)=s(t-\tau_{0})+w(t)$$

Substituting this into Equation (7), the correlator output becomes:

(10) $$\delta_{i}≜c_{s\hat{s}}(\tau_{i})+c_{w\hat{s}}(\tau_{i})$$

where:

(11) $$c_{s\hat{s}}(\tau_{i})=\frac{1}{T}\int_{(k-1)T}^{kT}e^{j\theta }s(t-\tau_{0}){\hat{s}}^{*}(t-{\hat{\tau }}_{0}-\tau_{i})dt$$

(12) $$c_{w\hat{s}}(\tau_{i})=\frac{1}{T}\int_{(k-1)T}^{kT}w(t){\hat{s}}^{*}(t-{\hat{\tau }}_{0}-\tau_{i})dt$$

The two terms are studied individually for their distinct characteristics. Equation (11) can be reformulated as follows, where inverse Fourier transform is applied:

(13) $$\begin{array}{cc} c_{s\hat{s}}(\tau_{i}) & =\frac{1}{T}\int_{(k-1)T}^{kT}e^{j\theta }s(t-\tau_{0}){\hat{s}}^{*}(t-{\hat{\tau }}_{0}-\tau_{i})dt\\ & =\frac{e^{j\theta }}{T}\int_{(k-1)T}^{kT}s(t-\tau_{0})\int_{-\beta_{r}/2}^{\beta_{r}/2}{\hat{\Phi }}^{*}(f)\;e^{-j2\pi f(t-{\hat{\tau }}_{0}-\tau_{i})}dfdt\\ & =\frac{e^{j\theta }}{T}\int_{-\beta_{r}/2}^{\beta_{r}/2}{\hat{\Phi }}^{*}(f)\;\int_{(k-1)T}^{kT}s(t-\tau_{0})e^{-j2\pi f(t-\tau_{0})}dt\;e^{-j2\pi f(\varepsilon -\tau_{i})}df\\ & =\frac{e^{j\theta }}{T}\int_{-\beta_{r}/2}^{\beta_{r}/2}{\hat{\Phi }}^{*}(f)\Phi (f)\;e^{-j2\pi f(\varepsilon -\tau_{i})}df\\ & =e^{j\theta }\int_{-\beta_{r}/2}^{\beta_{r}/2}X_{s,\hat{s}}(f)\;e^{-j2\pi f(\varepsilon -\tau_{i})}df \end{array}$$

where $X_{s,\hat{s}}$(*f*) is the cross-spectrum defined in Equation (5), which is of the essence to support unmatched receiving. The spectrum-based representation facilitates the evaluation under different front-end bandwidth, which is commonly conducted in the recent researches.

Similarly, by utilizing the properties of the zero-mean Gaussian process of noise and interference *w*(*t*), the variance of Equation (11) can be obtained:

(14) $$\mathrm{Var}(c_{w\hat{s}})=\frac{C_{s}}{T}\int_{-\beta_{r}/2}^{\beta_{r}/2}G_{w}(f)G_{\hat{s}}(f)df$$

where *G_w_*(*f*) is the power spectral density of the noise and interference *w*(*t*). It can also be derived that the modulus of correlator output │*δ_i_*│ has Rician distribution. According to Equations (11) and (12), the signal to noise and interference ratio (SNIR) can be obtained as:

(15) $$\rho (\tau_{i})=\frac{{\left|c_{s\hat{s}}(\tau_{i})\right|}^{2}}{\mathrm{Var}(c_{w\hat{s}})}=\frac{TC_{s}{\left|\int_{-\beta_{r}/2}^{\beta_{r}/2}e^{-j2\pi f(\varepsilon -\tau_{i})}X_{s,\hat{s}}(f)df\right|}^{2}}{\int_{-\beta_{r}/2}^{\beta_{r}/2}G_{w}(f)G_{\hat{s}}(f)df}$$

Consider the prompt branch $\delta_{P}$, where $\tau_{P}$ = 0, then Equation (15) can be used to evaluate the performance of acquisition and data demodulation, where unmatched receiving is supported.

In stable tracking where the random error of TOA estimation caused by noise and interference can be treated as zero, there is always *e* >> 0. Then moving the *TC_s_* to the denominator from Equation (15), at $\tau_{P}$ = 0, the first decoupled evaluation criterion is provided as:

(16) $$\eta_{\mathrm{mis}}=\frac{\rho (\tau_{P})}{TC_{s}}$$

where *η*_mis_ is defined as the correlation loss under unmatched receiving, used to characterize the performance of acquisition and demodulation. Different from the traditional evaluation criterion for acquisition in [10,11] and the new unmatched criterion Equation (15), Equation (16) decoupled from the integration duration *T* and average signal power *C_s_*. In addition, when only WGN exists in *w*(*t*), *η*_mis_ is further decoupled from the WGN strength *N*_0_ or *C_s_*/*N*_0_. Therefore, Equation (16) can better describe the performance of tracking without the effect of the receiver and environment. 

As a special case, when the local signal is exactly conjugated with the received signal, Equations (15) and (16) degenerates to the existing evaluation criterion for acquisition provided in [10,11]. In this case, it explains the power loss caused by band limiting, while result Equation (16) considers the factors of band limiting and local code mismatch.

### 4.2. Formulation and Statistical Properties of Discriminator Output

This subsection provides a derivation of the discriminator output, which is the foundation to estimate the error of pseudorange measurement. Based on the previously obtained correlation and I&D output Equation (10), the output of the early minus late power discriminator shown in Figure 1 can be expanded as:

(17) $$e(\varepsilon )=|\delta_{E}{|}^{2}-|\delta_{L}{|}^{2}\;=\;|c_{s\hat{s}}(\Delta /2){|}^{2}-|c_{s\hat{s}}(-\Delta /2){|}^{2}$$

Considering the random error caused by interference and Gaussian white noise, in stable tracking, the TOA estimation error is approximate to zero ε ≈ 0. By using Equation (17), the loop gain of e(ε) can be obtained after tedious derivation as:

(18) $$K={\frac{de(\varepsilon )}{d\varepsilon }|}_{\varepsilon \to 0}=8C_{s}^{2}\pi \int_{-\beta_{r}/2}^{\beta_{r}/2}X_{s,\hat{s}}(f)\cos(\pi f\Delta )df\;\times \int_{-\beta_{r}/2}^{\beta_{r}/2}fX_{s,\hat{s}}(f)\sin(\pi f\Delta )df$$

Note that the cross-spectrum $X_{s,\hat{s}}$(*f*) is also used here. Then the variance of TOA estimation at the output of correlator is obtained as:

(19) $$\begin{array}{rr} \mathrm{Var}(\varepsilon )=\frac{\mathrm{Var}\{e(\varepsilon )\}}{K^{2}} & =\frac{8}{K^{2}T}{\left(\int_{-\beta_{r}/2}^{\beta_{r}/2}X_{s,\hat{s}}(f)\cos(\pi f\Delta )df\right)}^{2}\times \int_{-\beta_{r}/2}^{\beta_{r}/2}G_{w}(f)G_{\hat{s}}(f)\sin^{2}(\pi f\Delta )df\\ & +\frac{2}{K^{2}T^{2}}[{\left(\int_{-\beta_{r}/2}^{\beta_{r}/2}G_{w}(f)G_{\hat{s}}(f)df\right)}^{2}-{\left(\int_{-\beta_{r}/2}^{\beta_{r}/2}G_{w}(f)G_{\hat{s}}(f)\cos(2\pi f\Delta )df\right)}^{2}] \end{array}$$

which is the open loop variance without being smoothed by the loop. Further, the variance of closed-loop TOA estimation error can be obtained with the relationship between the open-loop and the closed-loop result [11]:

(20) $$\sigma_{\mathrm{close}}^{2}(\varepsilon )=\mathrm{Var}(\varepsilon )\;2B_{L}T(1-0.5B_{L}T)$$

where *B_L_* is the one-sided equivalent noise bandwidth of the tracking loop, *T* is the integration duration where $0\leq B_{L}T\leq 0.5$ is satisfied, as previously defined in the model. 

Two assumptions are used here. First, let the early-late space ∆ tend to zero, which would obtain the lower bound of the code tracking error. The bound is strict in the sense that well-designed receiver can achieve competitive tracking accuracy [18]. Second, the noise and interference term *w*(*t*) is expanded into two terms as Equation (8), facilitating the study of the influence of noise and interference distinctly. Then Equation (20) becomes:

(21) $$\begin{array}{cc} \sigma_{\Delta \to 0}^{2}(\varepsilon ) & =\frac{B_{L}(1-0.5B_{L}T)}{{\left(2\pi \right)}^{2}}\left[\frac{\int_{-\beta_{r}/2}^{\beta_{r}/2}f^{2}G_{\hat{s}}(f)df}{\frac{C_{s}}{N_{0}}{\left(\int_{-\beta_{r}/2}^{\beta_{r}/2}f^{2}X_{s,\hat{s}}(f)df\right)}^{2}}+\frac{C_{\iota }}{C_{s}}\frac{\int_{-\beta_{r}/2}^{\beta_{r}/2}f^{2}G_{\iota }(f)G_{\hat{s}}(f)df}{{\left(\int_{-\beta_{r}/2}^{\beta_{r}/2}f^{2}X_{s,\hat{s}}(f)df\right)}^{2}}\right]\\ & \times \left[1+\frac{\int_{-\beta_{r}/2}^{\beta_{r}/2}G_{\hat{s}}(f)df}{\frac{C_{s}}{N_{0}}T{\left(\int_{-\beta_{r}/2}^{\beta_{r}/2}X_{s,\hat{s}}(f)df\right)}^{2}}+\frac{C_{\iota }}{C_{s}}\frac{\int_{-\beta_{r}/2}^{\beta_{r}/2}G_{\iota }(f)G_{\hat{s}}(f)df}{T{\left(\int_{-\beta_{r}/2}^{\beta_{r}/2}X_{s,\hat{s}}(f)df\right)}^{2}}\right] \end{array}$$

Equation (21) provides a low bound for the code tracking error evaluation, which considers the effect of unmatched receiving by using the cross-spectrum term $X_{s,\hat{s}}$ in the denominator. Also, derivation shows that the local spectrum $G_{\hat{s}}$ appears in the numerator, rather than cross-spectrum $X_{s,\hat{s}}$, which is confused in the previous work [12].

It should be noted that the result of Equation (21) is also compatible with the early minus late (E-L) discriminator, by discarding the terms after the multiplication sign, which depicts the squared loss caused by the non-coherent discriminator. Accordingly, the variance of the TOA estimation for the E-L discrimination is achieved by the first line of Equation (21).

Also, the results of Equation (21) can hold for matched receiving, with the local spectrum $G_{\hat{s}}$ and cross-spectrum $X_{s,\hat{s}}$ substituted with the received spectrum *G_s_*(*f*).

### 4.3. Multipath Effects under Unmatched Receiving

In this subsection, the effect of multipath under unmatched receiving is discussed. Consider a basic and representative model with a one-path specular reflection [18]. To evaluate the average influence of multipath, the average range error envelope [18] is utilized, defined as:

(22) $$\Gamma (\delta_{m})=\frac{1}{\delta_{m}}\int_{0}^{\delta_{m}}\left(\underset{\phi }{\max}\varepsilon (\alpha ,u,\phi )-\underset{\phi }{\min}\varepsilon (\alpha ,u,\phi )\right)du$$

where *α* is the multipath-to-direct ratio (MDR), *δ_m_* is the relative delay of multipath signal compared with the direct signal, ϕ is the relative carrier phase of multipath signal compared with the direct signal. In Equation (22), ε(α,u,ϕ) is the error caused by zero crossing bias of discriminator curve. The discriminator curve in the present of multipath is given by [19]:

(23) $$\begin{array}{cc} D(\tau )= & R_{g\overset{⌢}{g}}^{2}\left(\tau +\frac{\Delta }{2}\right)-R_{g\overset{⌢}{g}}^{2}\left(\tau -\frac{\Delta }{2}\right)+\alpha^{2}\left[R_{g\overset{⌢}{g}}^{2}\left(\tau +\frac{\Delta }{2}-\delta_{m}\right)-R_{g\overset{⌢}{g}}^{2}\left(\tau -\frac{\Delta }{2}-\delta_{m}\right)\right]\\ & +2\alpha \cos\phi \cdot \left[R_{g\overset{⌢}{g}}^{2}\left(\tau +\frac{\Delta }{2}\right)\cdot R_{g\overset{⌢}{g}}^{2}\left(\tau +\frac{\Delta }{2}-\delta_{m}\right)-R_{g\overset{⌢}{g}}^{2}\left(\tau -\frac{\Delta }{2}-\delta_{m}\right)\right] \end{array}$$

where $R_{g\overset{⌢}{g}}$ is the cross-correlation function between received signal and local despreading signal.

As can be seen, both the multipath component and the mismatch of local signal and received signal together influence the zero-crossing point of DLL discriminator curve, thus resulting in different characteristics in the ranging error than the multipath effect under matched receiving.

To give an overall evaluation of the multipath effect in unmatched receiving, simulation of the multipath resistance performance is provided for ACE-BOC and MBOC signals in Section 6.2.4 and Section 7.2.4.

## 5. Decoupled Criteria for GNSS Code Tracking Evaluation

### 5.1. Anti-Interference Rate

The unmatched code tracking evaluation criterion Equation (21) is coupled with numerous factors. In order to study the influence of one factor, it needs a hypothetical assumption of other factors. While in the signal design, it is not enough to consider merely one hypothetical assumption, but as many as possible to reflect the actual case. However, traversing all the possible value of factors exaggerates the calculation, as well as making the useful information overwhelmed by the influence of numerous factors. Therefore, a concise evaluation criterion considering only essential factors is necessary.

To study the effect of interference in the theoretical evaluation, a criterion with similar physical meaning to the quality factor, or Q factor is needed [18]. For this purpose, part of the coefficients of the signal to interference ratio (SIR) is obtained from the variance of ranging error Equation (21), and its root square is shown in Equation (24) with the unit of Hz^–1^, defined as the *anti-interference rate*:

(24) $$\nu =\sqrt{\frac{X_{\iota \hat{s}}}{\varpi^{2}\beta_{\hat{s}}^{2}}}$$

where:

(25) $$\varpi =\frac{\int_{-\beta_{r}/2}^{\beta_{r}/2}f^{2}X_{s,\hat{s}}(f)df}{\sqrt{\int_{-\beta_{r}/2}^{\beta_{r}/2}f^{2}G_{\hat{s}}(f)df}}$$

(26) $$\beta_{\hat{s}}=\sqrt{\int_{-\beta_{r}/2}^{\beta_{r}/2}f^{2}G_{\hat{s}}(f)df}$$

(27) $$X_{\iota \hat{s}}=\int_{-\beta_{r}/2}^{\beta_{r}/2}f^{2}G_{\iota }(f)G_{\hat{s}}(f)df$$

The anti-interference rate Equation (24) represents the derivative of the interference strength, in other words, it represents the signal’s ability to resist a certain kind of interference. Except for the interference, other essential influencing factors of receiving bandwidth, local despreading waveform, are also necessary to be considered in the criterion. Meanwhile, some external factors represent less of the ability of signal to resist interference, such as interference strength, WGN strength, and other receiver implementation parameters, are not included in the criterion. Thus, the anti-interference rate is a decoupled criterion, which can better represent the inherent performance of the received signal under interference. It also supports the unmatched receiving.

It should be noted that, although Equation (24) seems like the normalization of the previous defined code tracking spectral separation coefficient (CT-SSC) [11], provided as Equation (27), further study shows that the anti-interference rate has substantial distinction from the code tracking SSC. First, the anti-interference rate Equation (24) considers the effect of unmatched receiving, which is not representing in the traditional CT-SSC [11] itself. Second, from the physical meaning aspect, the traditional CT-SSC itself depicts the spectral separation between the interference and received signal, while the anti-interference rate is influenced by the spectrum of interference, received signal and also local despreading signal, thus representing an overall effects of interference on the code tracking. From this point of view, it should also be distinguished with an evaluation criterion named effective *C*/*N*_0_, which is also defined in [11]. Effective *C*/*N*_0_ reflects the effect of interference on the correlator output SNIR, thereby it is a useful criterion for evaluation of acquisition performance. 

Moreover, the intermediate results Equation (25), Equation (26) have specific meaning and are available for tracking performance evaluation.

### 5.2. Equivalent Gabor Bandwidth

Equation (25) is defined as the ‘Equivalent Gabor bandwidth’ in our previously published paper [14], used for code tracking evaluation under unmatched receiving. Literally, it can be seen as a generalization of the original Gabor bandwidth [10], in order to support unmatched receiving. It reflects the capability of a signal to resist WGN in code tracking. From Equation (25) we can see that performance is only influenced by the local signal, mismatch of local despreading waveform and receiving bandwidth. The cross-spectrum $X_{s,\hat{s}}$(*f*) in Equation (21) depicts the mismatch between the local despreading signal and the received signal, which is not considered in the previous work.

Also, the generalized evaluation criterion equivalent Gabor bandwidth Equation (25) can degenerate to the original Gabor bandwidth, as defined in Equation (26), which is for the local signal $\hat{s}$. Therefore, with the proposed signal evaluation criteria, the low bound of code tracking error is simplified as:

(28) $$\sigma_{\text{NELP,LB}}^{2}=\frac{B_{L}(1-0.5B_{L}T)}{{\left(2\pi \right)}^{2}}\left(\frac{1}{\frac{C_{s}}{N_{0}}\varpi^{2}}+\frac{C_{\iota }}{C_{s}}\nu^{2}\right)\times \left(1+\frac{\zeta }{T}\right)$$

where ζ is the squared loss factor:

(29) $$\zeta =\frac{1}{\frac{C_{s}}{N_{0}}\eta_{n}}+\frac{1}{\frac{C_{\iota }}{N_{0}}\eta_{\iota }}$$

*η_n_* is the correlation loss derived from Equation (16) with only WGN existing, and *η_ι_* is the correlation loss derived from Equation (16) non-white noise interference *C_ι_G_ι_*(*f*). The squared loss factor ζ is relatively small compared with the integration duration *T* in most cases, except the cases of extremely low SNR and strong interference.

From the code tracking error bound Equation (28) we can see that, by observing the unmatched criteria equivalent Gabor bandwidth ϖ and the anti-interference rate ν, one can get the essential abilities to resist the WGN and interference of a signal, regardless of the specific hypothetical assumption of SNR or SIR, making the results more universal. Therefore, the evaluation results are more persuasive in the comparing of the inherent performance among different signals. 

It should be noted that, the evaluation criteria defined in Equations (24) and (25) consider the mismatch between local despreading code chip waveform and the received spreading code chip waveform. By letting the local spectrum match the received signal spectrum, the proposed evaluation criteria in Equations (24) and (25) are also available for the evaluation of matched receiving. 

In the following two sections, the proposed criteria are used to evaluate ACE-BOC and MBOC signals, from which some instructive suggestions are derived for signal design and receiver configuration. As a summary of the mathematical model and evaluation theory, the important parameters used in this paper are provided in a nomenclature list.

## 6. The Evaluation of Unmatched Receiving of ACE-BOC

### 6.1. ACE-BOC Overview and Receiving Strategies

The Asymmetrical Constant Envelope BOC(ACE-BOC) [19,20,21] is a flexible constant envelope multiplexing or modulation technique. An ACE-BOC signal is a constant envelope signal composed of no more than four baseband signal components, which are located at two central frequencies and allocated with arbitrary power. The ACE-BOC multiplexed signal is denoted as ACE-BOC(*m*, *n*, [*P*_UI_,*P*_LI_,*P*_UQ_,*P*_LQ_]), where the *m* stands for the spreading code rate *f_c_* = *m* × 1.023 MHz, and *n* stands for the subcarrier rate *f_sc_* = *n* × 1.023 MHz. *P_wv_* stands for the relative power of the signal component modulated to the upper or lower sideband, denoted by w∈{U,L}, with the in-phase or quadrature-phase, denoted by v∈{I,Q}. The generating formula of ACE-BOC(*m*, *n*, [*P*_UI_,*P*_LI_,*P*_UQ_,*P*_LQ_]) signal is provided in [21,22].

As a dual-frequency, four-components multiplexed signal, ACE-BOC supports multiple receiving strategies with different level of performance, including a matched receiving and two unmatched receiving strategies [22]:

- Matched Receiving

The matched receiving can make full use of the wideband signal characteristics. However, wideband receiving requires large front-end bandwidth and higher complexity in generating the exactly matched local despreading code [22], which is hard to afford for some of the receivers.

- Independent Matched Receiving (IMR)

In IMR, the local baseband despreading replica is adapted to match one of the composite signal components, including the PRN code and subcarrier. For example, ${\hat{s}}_{\mathrm{UQ}}\left(t\right)=s_{\mathrm{UQ}}\left(t\right)\left[sc_{2}\left(t\right)-jsc_{2}\left(t-\frac{T_{sc}}{4}\right)\right]$ is the baseband local replica for the upper sideband Q-channel. Triple-loop tracking [5] or other techniques can be applied for the subcarrier tracking for IMR.

- BPSK-like Receiving (BLR)

BLR treats one of the ACE-BOC components as a BPSK signal, so as to directly work with general BPSK receivers. The receivers set the local carrier frequency at the center of one of the main lobes of the dual-frequency complex signal, i.e., *f_sc_* or –*f_sc_* under zero intermediate frequency assumption [23]. It can be noticed that no subcarrier is needed to be generated in the BLR, which enables the receiver to operate at a much lower sampling rate. Accordingly, BLR is widely used by low-cost consumer market receivers.

The unmatched reception of ACE-BOC signals has a wide range of applications due to its low-complexity. By using the proposed evaluation methods, the performance under the three receiving strategies and various influencing factors can be illustrated in the next subsections, providing a comprehensive understanding to the signal design, as well as constructive suggestions to receiver configuration.

### 6.2. Performance Evaluation

#### 6.2.1. Acquisition Performance Evaluation

**Figure 2:**
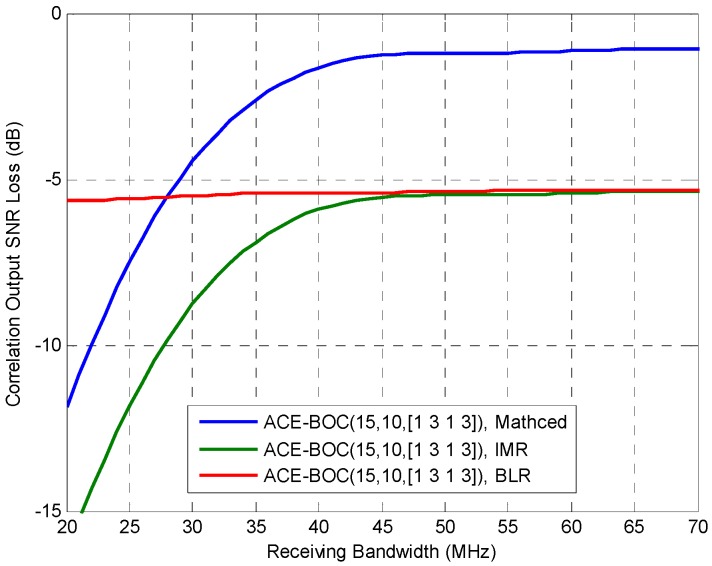
The correlation output SNIR loss of ACE-BOC(15,10,[1,3,1,3]), where the central frequency of receiving of Matched receiving and IMR is at *f*_0_, and the central frequency of BLR is at *f*_0_ – *f_sc_*. The IMR and BLR receives the component *s*_LQ_, whose power ratio among the useful transmitting power is 3/8.

For a CEM signal as ACE-BOC, different acquisition performance can be achieved by different processing modes. In Figure 2, an evaluation of acquisition performance is conducted for ACE-BOC(15,10,[1,3,1,3]) by utilizing Equation (15), where three receiving strategies are considered, which are matched receiving, IMR and BLR. The matched receiving makes full use of the wide-band ACE-BOC signal. IMR and FMR process the component *s*_LQ_, which is modulated on the lower band and quadrature phase. In this case, *s*_LQ_ occupies 3/8 of the total transmission power. Thus, the central frequency of IMR and matched receiving is at *f*_0_, which is the central frequency of the wideband ACE-BOC spectra. The central frequency of BLR is *f*_0_ − *f_sc_*, at the center of lower main lobe. To explicitly show the evaluation scenario of ACE-BOC signal, the related parameters are enumerated in Table 1.

As can be seen from Figure 2, when the receiving bandwidth is smaller than 28 MHz, BLR achieves the smallest correlation SNR loss, indicating that BLR has the best acquisition performance at small receiving bandwidth. When the receiving bandwidth is higher than 28 MHz, matched receiving achieves better acquisition performance than IMR and BLR, whose correlation SNR loss shows no obvious distinction, because both BLR and IMR make use of a signal component with 3/8 of the total power of ACE-BOC signal. Also, compared with IMR, BLR performs better when the receiving bandwidth is smaller than 46 MHz, which is close to the bandwidth covering two main lobes of ACE-BOC signal. 

From the results above, considering the performance and complexity of the three receiving modes, instructive advice can be obtained for receivers. It can be seen that in terms of acquisition performance, IMR is not recommended because it shows no obvious advantage over the BLR at any bandwidths, while IMR requires more receiver complexity than BLR.

#### 6.2.2. Tracking Performance under WGN

**Figure 3:**
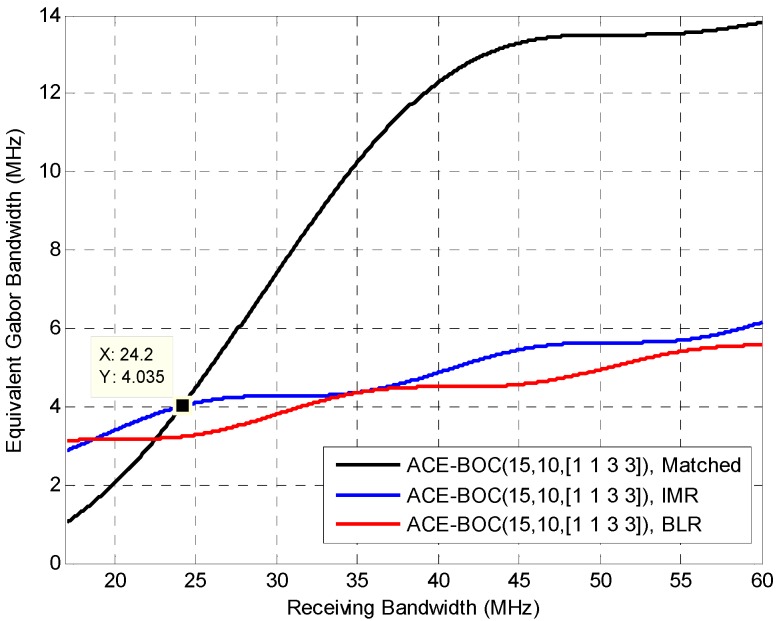
Equivalent Gabor bandwidth of ACE-BOC, where the central frequency of receiving of Matched receiving and IMR is at *f*_0_, and the central frequency of BLR is at *f*_0_ − *f_sc_*. The IMR and BLR receives the component *s*_LQ_, whose power ratio among the useful transmitting power is 3/8.

To study the code tracking performance under WGN, Figure 3 shows the equivalent Gabor bandwidth of ACE-BOC(15,10,[1,1,3,3]) under the aforementioned three receiving strategies. IMR and BLR receives the lower sideband, Q-channel signal component *s*_LQ_. The receiving configuration can be referred to Table 1.

Different from the code tracking error Equation (28), which is also used to evaluate the code tracking performance, the equivalent Gabor bandwidth decoupled from the integration duration, SNR, interference strength, making the evaluation of the signal less affected by the specific working scenario of receivers. 

As shown in Figure 3, when the receiving bandwidth exceeds 24.2 MHz, matched receiving shows a notable advantage over the IMR and BLR. The equivalent Gabor bandwidth of IMR is slightly larger than BLR, because the IMR uses a square waveform subcarrier which is matched with the received signal, while BLR uses a sinusoidal waveform subcarrier which employs only the energy on the 1st harmonics of the subcarrier frequency. 

From the results shown in Figure 3, useful information can be obtained to guide the receiving strategies. First, it should be noted that when the receiving bandwidth is about 35 MHz, there is no need to apply the more complicated IMR, as BLR can achieve competitive performance with IMR. Also, when the receiving bandwidth can be increased to obtain better ranging accuracy, the increment should refer to the slope of curves shown in the Figure 3. For instance, when the original bandwidth is 25 MHz for BLR, increasing 10 MHz bandwidth brings a notable promotion of 1.08 MHz in Equivalent Gabor bandwidth. While, when the original bandwidth is 35 MHz, the benefit in Equivalent Gabor bandwidth by increasing 10 MHz of bandwidth is only 0.214 MHz.

#### 6.2.3. Tracking Performance under Interference

**Figure 4:**
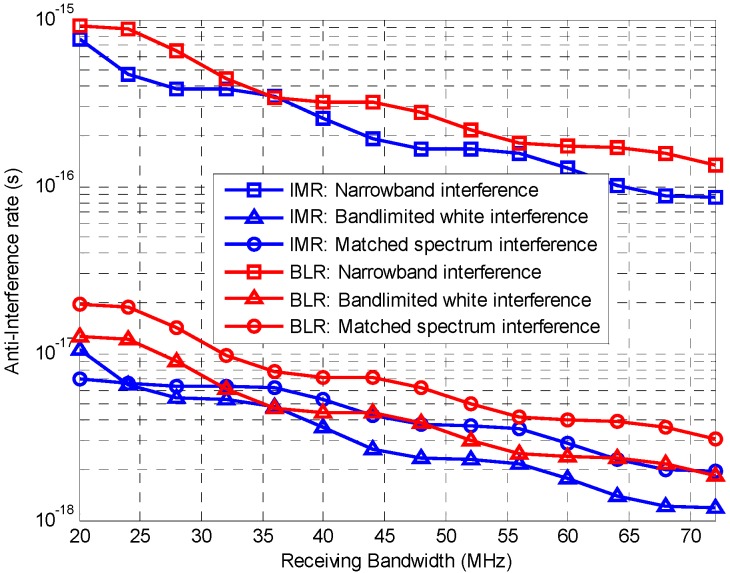
Anti-interference rate of ACE-BOC under three kinds of interference: narrowband interference, bandlimited interference and matched spectrum interference. Receiving strategies are IMR and BLR for component *s*_LQ_. Larger value means more vulnerability to such interference.

The anti-interference rate *n* proposed in Equation (24) can be used to evaluate the performance of code tracking under interference, that larger value of *n* means the code tracking is more vulnerable to such interference. The anti-interference rate for ACE-BOC(15,10,[1,1,3,3]) is shown in Figure 4, with the receiving strategies of IMR and BLR for the lower sideband, Q-channel signal component *s*_LQ_, under three kinds of interference. The narrowband interference has a bandwidth of 10 kHz and a center frequency 1 MHz apart from the main lobe center of *s*_LQ_, and the wideband interference has double-side band width of 5 MHz. Matched spectrum interference has the spectrum The interference configuration is listed in Table 2. As an advantage of the decoupling of the anti-interference rate, there is no need to assume the interference strength, integration duration, loop bandwidth, etc.

As can be seen in Figure 4, firstly, in general the anti-interference rate decreases as the receiving bandwidth increases, indicating that the interference resistance ability is improved with larger bandwidth. Secondly, for both IMR and BLR of ACE-BOC, narrowband interference is much serious than the bandlimited and matched spectrum interference, under the given conditions, implying that in a challenging environment, narrowband interference resistance techniques should be the primary consideration to improve the overall positioning performance.

In terms of the receiving configuration, evaluation results show that IMR achieves better anti-interference performance at most bandwidths. However, when the receiving bandwidth is about 36 MHz, IMR shows no advantage over BLR under narrowband interference and bandlimited white interference. With the proposed evaluation criterion, it is able to provide the inherent performance of signal, and support various unmatched receiving.

#### 6.2.4. Multipath Resisting Performance

**Figure 5:**
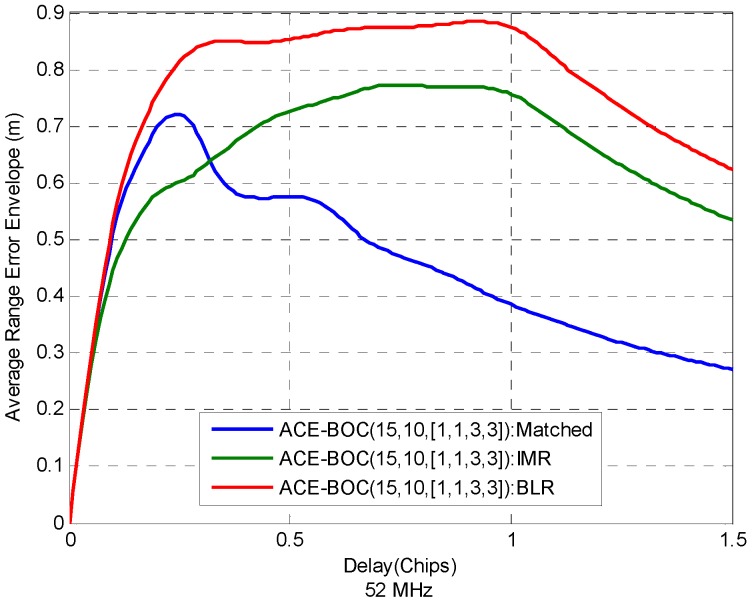
Average range error envelope of ACE-BOC(15,10,[1,1,3,3]), under matched receiving, IMR and BLR. The double-sided receiving bandwidth 52 MHz. MDR is −5 dB.

For ACE-BOC, as mentioned, different receiving strategies result in different shapes in cross-correlation function, which also influence the ranging performance under multipath. Figure 5 shows the average range error envelope Equation (22) of ACE-BOC(15,10,[1,1,3,3]) under three receiving modes, with 52 MHz bandwidth when MDR is −5 dB. The IMR and BLR receive the component *s*_LQ_. The receiver configuration can be seen in Table 1.

It can be seen that in wideband receiving of 52 MHz, as shown in Figure 5, the multipath resistance performance ranks as matched > IMR > BLR when the relative delay of the multipath over the direct signal is larger than 0.32 chips. When the relative delay is smaller than 0.32 chips, however, the multipath resistance of matched receiving is even larger than IMR. It indicates that IMR works better at short-delay multipath scenarios.

To summarize, receiving strategy selection for a multi-resolution signal like ACE-BOC is a balance among multiple performance aspects and complexities. BLR requires the least complexity and achieves advantageous acquisition performance with narrow receiving bandwidth which is no more than 28 MHz. When higher complexity in receiver is supported, including wider front-end bandwidth and more complicated local despreading waveform generator, IMR can trade the complexity to notable enhancement in accuracy than BLR. Further, if the most complex matched receiving can be implemented, it is possible to make full use of the wideband ACE-BOC signal, thus the optimum performance can be obtained. Details of the trade-off between performance and receiving complexity can be observed from the evaluation results, which provides a reference in receiving strategies selection.

## 7. The Evaluation of BOC-Like Receiving of MBOC

### 7.1. MBOC Overview and Receiving Strategies

In the modernized GNSS, to help promote the availability of positioning service for civil use, interoperation should be obtained between different GNSS. Multiplexed Binary Offset Carrier (MBOC) [24,25] is a candidate scheme for the L1 band civil signal, which is commonly accepted by GPS, Galileo and BDS as interoperation signal. MBOC defines that the signal spectrum should satisfy:

(30) $$G(f)=\gamma G_{\mathrm{BOC}(m,n)}+(1-\gamma )G_{\mathrm{BOC}(n,n)},\;\;m>n$$

A MBOC(*m,n,γ*) signal comprises two BOC modulated signals: a wideband BOC(*m*, *n*) and a narrowband BOC(*n*, *n*), with *m > n*, and *γ* is a power coefficient with 0 ≤ *γ* ≤ 1. In practice, since the spectral characteristic Equation (30) is the only limitation for MBOC, various implementations can be used to generate the MBOC signal, including Composed Binary Offset Carrier (CBOC), Time-division Multiplexed Binary Offset Carrier (TMBOC), Quadrature Multiplexed BOC (QMBOC) [26], etc.

As a composite signal, MBOC enables multiple receiving strategies, where different levels of accuracy can be obtained with the corresponding receiver complexity. Matched receiving of MBOC enables the optimum received performance. The unmatched receiving is more widely used, generally referred as BOC-like receiving, where only the low-order BOC(*n*, *n*) component is processed in the receiver. Therefore, the local replica generation of BOC-like receiving is less complex than the matched way. Take MBOC(6,1,1/11) for example, matched receiving requires the bandwidth to be more than 14 × 1.023 MHz, while narrowband BOC-like receiving requires a bandwidth of only 4 × 1.023 MHz.

It should be noted that discarding the high-order BOC(*m*, *n*) component does not cause significant power loss. Because in MBOC(6,1,1/11), the low-order BOC(1, 1) accounts for 10/11 of the total transmitting power, ensuring the quality of primary positioning service for the vast unmatched receivers. 

Therefore, signal performance evaluation for the various receiving strategies of MBOC becomes essential in recent years for its widespread use. Reference [14] provides analysis of the unmatched equivalent Gabor bandwidth in Equation (25), which is available in the evaluation of code tracking accuracy, anti-multipath performance of a signal, along with its receiving strategy. Further, in this work, the performance evaluation criterion of non-white noise interference resistance is provided in Equation (24), which facilitates the signal analysis and comparison in multiple dimensions.

### 7.2. Performance Evaluation

#### 7.2.1. Acquisition Performance

As mentioned in the previous subsection, many specific implementations satisfy the spectral constraint Equation (30) of MBOC, including TMBOC, QMBOC, CBOC+, CBOC−, which are evaluated in this section. Those specific MBOC implementations all support the BOC_11_-like unmatched receiving.

**Figure 6:**
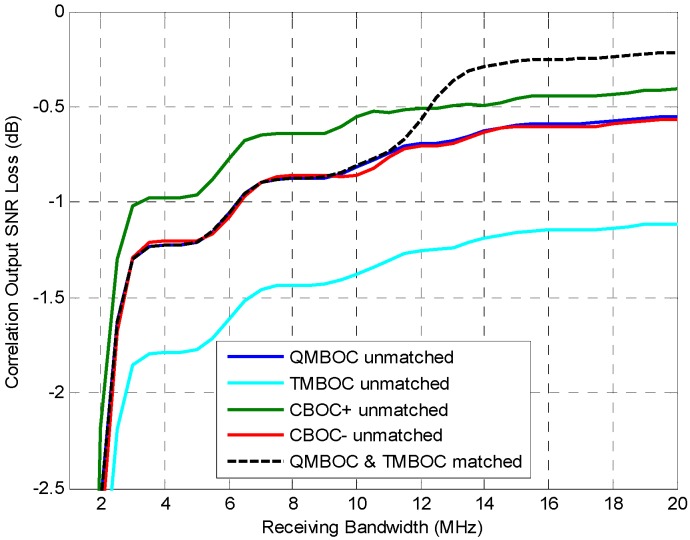
The correlation output SNIR loss of multiple implementation of MBOC(6,1,1/11) signals, under matched receiving and BOC_11_-like unmatched receiving. It can be used to characterize the acquisition performance of MBOCs.

The acquisition performance for the several MBOC signals can be compared by using the unmatched correlator output SNIR loss Equation (16), which supports unmatched receiving, as shown in Figure 6. Among the four MBOCs, generally, the rank of the acquisition performance is CBOC+ > CBOC− = QMBOC > TMBOC for the unmatched receiving. In addition, the dashed line in Figure 6 shows the performance of matched received QMBOC or TMBOC for reference, whose acquisition performances are the same in matched receiving [27] because an identical spectrum is achieved. The dashed curve ramps up as the receiving bandwidth increases, because a higher proportion of power is acquired within the receiving band, which is 93.5% of the transmitting power at the receiving bandwidth 14 MHz, and 95% at 20 MHz.

It is worth noting that the matched QMBOC and TMBOC have even worse acquisition performance than unmatched CBOC+ when the bandwidth is less than 12.1 MHz. Also, the matched QMBOC and TMBOC achieve competitive performance with the unmatched CBOC− and unmatched QMBOC when the bandwidth is less than 10.5 MHz. This demonstrates that for those receivers with not large enough front-end bandwidth, it is not beneficial to employ the matched receiving with an additional complexity rather than the unmatched receiving.

#### 7.2.2. Tracking Performance under WGN

**Figure 7:**
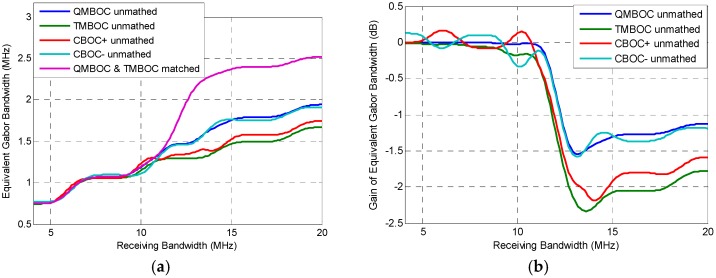
Equivalent Gabor bandwidth of for implementations of MBOC(6,1,1/11), under matched receiving and BOC_11_-like unmatched receiving, representing the code tracking performance. (**a**) Equivalent Gabor bandwidth; (**b**) Additional equivalent Gabor bandwidth gain, compared with matched QMBOC and TMBOC, which is achieved by Equation (31).

The comparison of code tracking performance of the implementations of MBOC can be provided by using the equivalent Gabor bandwidth Equation (25), as shown in Figure 7a, where the carrier to noise ratio is 40 dB. Figure 7b further shows the additional equivalent Gabor bandwidth gain, where four unmatched receiving methods are compared with the matched receiving of TMBOC and QMBOC, which is obtained by

(31) $${\mathrm{Gain}}^{\left(i\right)}{=10\log}_{10}\left(\frac{\varpi_{\mathrm{unmatched}}^{\left(i\right)}}{\varpi_{\mathrm{matched}}}\right),\;\;i\in \{\mathrm{QMBOC},\;\mathrm{TMBOC},\;\mathrm{CBOC}+,\;\mathrm{CBOC}-\}$$

where $\varpi^{\left(i\right)}$ stands for the equivalent Gabor bandwidth, superscript *i* indicates a modulation among the four implementations of MBOC. Benefitting from the perspective of gain, more details can be seen from Figure 7b.

As shown in Figure 7a,b, when the bandwidth is smaller than 11 MHz, the code tracking performance obtained by the matched receiving and unmatched receiving are almost similar. After 11 MHz, the matched receiving shows clear advantage towards the unmatched receiving. Comparing the four implementation of MBOC, when unmatched BOC_11_-like receiving is applied, the rank of code tracking performance is roughly QMBOC >> CBOC− > CBOC+ > TMBOC. From the above, constructive results can be obtained for the receiving strategies, whereby matched receiving is not recommended for the low-end receivers with small front-end bandwidth. It is also constructive to the signal transmitting side that QMBOC provides better acquisition and tracking performance than TMBOC. Comparing the two CBOCs, the BOC_11_-like received CBOC+ achieves better acquisition performance, while CBOC− achieves better tracking performance. Results show that, by using the evaluation criterion which supports both the matched receiving and unmatched receiving, it is convenient and effective in providing comparison of the different signal designs and receiving strategies.

#### 7.2.3. Anti-Interference Performance

**Figure 8:**
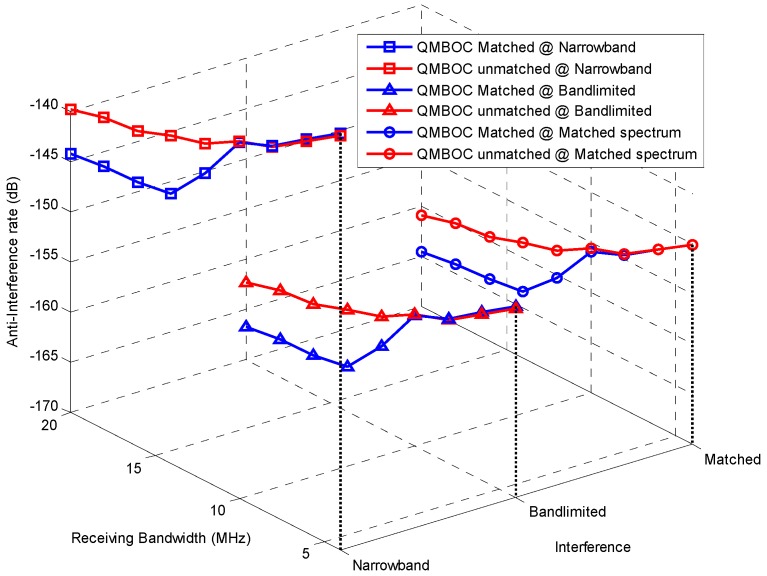
Anti-interference rate of QMBOC(6,1,1/11) with respect of receiving bandwidth, under the matched receiving and BOC_11_-like unmatched receiving. Three types of interferences are considered, including narrowband interference, bandlimited Gaussian interference and matched spectrum interference, of which the parameters setting of this figure is provided in Table 2.

For the assessment of the code tracking performance under interference, the anti-interference rate Equation (24) can be used, where a larger value means higher sensitivity to a certain kind of interference. The anti-interference performance is evaluated for QMBOC(6,1,1/11) in Figure 8, with respect to the receiving bandwidth, under three types of interference: narrowband, bandlimited and matched-spectrum. The parameters of interference are listed in Table 2. The receiving and despreading strategies include matched receiving and BOC_11_-like receiving.

From Figure 8 one can see, firstly, under a certain kind of interference, the anti-interference rates *n* of matched and unmatched receiving almost coincide at narrow receiving bandwidth of no more than 9.32 MHz. After 9.32 MHz, matched receiving shows an observable advantage towards the BOC_11_-like unmatched receiving, with a difference of 4.35–5.17 dB for the three interference types at the typical wideband receiving bandwidth of 15 MHz. Both BOC(1,1) unmatched users and wideband matched users can achieve good performance from the MBOC signal design. Secondly, from Figure 8 it is easy to compare the impacts caused by different types of interference. Narrowband interference that is near the main lobe of the signal is the most serious, and the bandlimited white noise and matched spectrum interference have almost similar impact on the tracking, under the given conditions. Moreover, further analysis can be conducted on the influencing factors of code tracking performance by observing the anti-interference rates, including the center frequency of the narrowband interference, the bandwidth of the bandlimited interference, etc.

Above all, by using the proposed anti-interference rate, the unmatched and matched tracking performance of QMBOC is provided under different bandwidth and interference scenarios, which provides useful guidance for the design of MBOC, as well as the anti-interference measures conducted by QMBOC receivers.

#### 7.2.4. Multipath Resisting Performance

**Figure 9:**
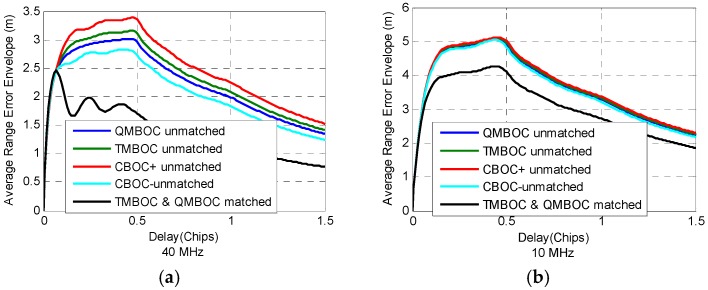
Average range error envelope of MBOC(6,1,1/11), under matched receiving and BOC_11_-like unmatched receiving. (**a**) Double-sided receiving bandwidth is 40 MHz; (**b**) Double-sided receiving bandwidth is 10 MHz. MDR is −5 dB.

In this subsection, a comparison of the multipath resistance ability of different MBOCs are provided, under unmatched BOC_11_-like receiving, which may help understand the instinct performance of different MBOC implementations. Figure 9 shows the average range error envelope Equation (22) of QMBOC, TMBOC, CBOC+ and CBOC− at receiving bandwidth 40 MHz and 10 MHz, where MDR is −5 dB.

As can be seen from the Figure 9a, when receiving bandwidth is 40 MHz, among the unmatched receiving of the four MBOCs, the multipath resistance ability is ranked as CBOC+ > QMBOC > TMBOC > CBOC−. Also, the matched receiving of QMBOC achieves better anti-multipath performance than the unmatched received MBOCs. When receiving bandwidth is 10 MHz, however, it shows no obvious difference between the four MBOCs under unmatched receiving. Matched receiving also shows the best anti-multipath performance at 10 MHz bandwidth.

Finally, it should be noted that in the previous signal evaluations by utilizing equivalent Gabor bandwidth and anti-interference rate for code tracking, we decoupled the evaluation criteria from external factors, such as the SNR, interference strength, receiver implementation details, etc. While the factors including mismatch of the despreading waveform are treated as an essential factor. This was done because the local despreading waveform is always selected according to the characteristics of the multi-components multiplexed signal. To reduce the receiving complexity, it is common to process a proportion of the components. For instance, the unmatched BOC_11_-like receiving of MBOC is achieved by discarding the BOC(6,1) term in the construction of the local replica. The unmatched BPSK-like receiving of high-order BOC or ACE-BOC is essentially achieved by ignoring the step-shaped subcarrier of the received signal in the local replica generation. Otherwise, if an unmatched receiving method is arbitrarily configured without the consideration of the structure of signal, it may deteriorate the received SNR seriously, thus affecting the operating of receiver. Therefore, the diverse unmatched receiving approach for the signal does not depart from the scope of the evaluation of the internal factors of signal.

## 8. Conclusions

This paper has provided a theoretical tracking performance evaluation method for unmatched receiving. Firstly, the mathematical model of the DLL in GNSS receivers has been generalized to consider the mismatch of the local despreading code chip waveform. Based on the unmatched tracking model, performance evaluation criteria for code tracking error have been derived, which are notable in the equivalent Gabor bandwidth and anti-interference rate. These criteria decouple from the less related factors and concentrate on the essential factors of the GNSS signal, which characterizes the inherent properties of a signal. Then, the proposed evaluation criteria have been applied to evaluate two split-spectrum signals ACE-BOC and MBOC. The signal performance of acquisition, tracking and interference resistance have been assessed and compared among different receiving strategies. Useful information can be derived by using the evaluation criteria, which is helpful for signal design and receiver configuration. 

## Figures and Tables

**Table 1:** Receiver configuration of the three receiving strategies of ACE-BOC, which is used in the evaluation in Section 6.2.

	Receiving Central Frequency(*f_r_*)	Receiving Components
BLR	*f*_0_ − *f_sc_*	*s*_LQ_
IMR	*f*_0_	*s*_LQ_
FMR	*f*_0_	Integral signal

**Table 2:** Interference parameters in the evaluation scenario. This configuration is effective in Section 6.2.3 and Section 7.2.3.

Central Frequency of Narrowband Interference	Bandwidth of Narrowband Interference	Bandwidth of Bandlimited Interference
*f_r_* ± 1 MHz ^1^	10 kHz	5 MHz

^1^$f_{r}$ stands for the receiving central frequency. For ACE-BOC, $f_{r}$ is specified in Table 1. For MBOC, $f_{r}$ is the central frequency of the double-sideband MBOC spectra, which is denoted as $f_{0}$.

## Nomenclature

$\beta_{\hat{s}}$	Gabor bandwidth.
ϖ	Equivalent Gabor bandwidth.
c(t)	DSSS waveform from the received signal.
$\hat{c}(t)$	DSSS waveform from the local despreading signal.
$f_{sc}$	Subcarrier frequency.
$f_{c}$	PRN chip rate, $f_{c}=1/T_{c}$.
$T_{c}$	Pulse width of c(t).
$\tau_{0}$	True value of TOA.
$\tau_{i}$	Fixed delay of correlators, i∈{E,P,L} stands for the branches of early, prompt and late.
n(t)	White Gaussian Noise (WGN).
$N_{0}$	Double-sided spectral density of WGN.
ι(t)	Non-white Gaussian interference.
ω(t)	Gaussian noise and interference.
$G_{\iota }(f)$	Normalized PSD of interference, satisfying $\int_{-\infty }^{+\infty }G_{\iota }(f)=1$.
$G_{s}(f)$	Normalized PSD of signal s(t), satisfying$\int_{-\infty }^{+\infty }G_{s}(f)=1$.
$C_{s}$	Average carrier power, defined on the infinite bandwidth.
$C_{s}/N_{0}$	Carrier to noise ratio.
$X_{s,\hat{s}}(f)$	Cross-spectrum between the received signal and the local signal.
$\eta_{\mathrm{mis}}$	Correlation loss under unmatched receiving, used to characterize the performance of acquisition and demodulation.
$X_{\iota \hat{s}}$	Code tracking spectral separation coefficient (CT-SSC).
$\sigma_{\text{NELP,LB}}^{2}$	Low bound of code tracking error, considering the unmatched despreading.
ν	Anti-interference rate.
$\Gamma (\delta_{m})$	Average range error envelope caused by multipath.

## References

[1] Yang C., Miller M., Nguyen T., Akos D. Generalized frequency-domain correlator for software GPS receiver: Preliminary test results and analysis. Proceedings of the 19th International Technical Meeting of the Satellite Division of the Institute of Navigation (ION GNSS 2006).

[2] Nunes F.D., Sousa F.M., Leitao J.M. (2007). Gating functions for multipath mitigation in GNSS BOC signals. IEEE Trans. Aerosp. Electron. Syst..

[3] Fishman P.M., Betz J.W. Predicting performance of direct acquisition for the M-code signal. Proceedings of the 2000 National Technical Meeting of the Institute of Navigation.

[4] Martin N., Leblond V., Guillotel G., Heiries V. BOC (x, y) signal acquisition techniques and performances. Proceedings of the 16th International Technical Meeting of the Satellite Division of the Institute of Navigation (ION GPS/GNSS 2003).

[5] Hodgart M.S., Blunt P.D. (2007). Dual estimate receiver of binary offset carrier modulated signals for global navigation satellite systems. Electron. Lett..

[6] Simon M.K., Omura J.K., Scholtz R.A., Levitt B.K. (1985). Spread Spectrum Communications; Vols. 1–3.

[7] Holmes J.K. Code tracking loop performance including the effects of channel filtering and Gaussian interference. Proceedings of the IAIN World Congress and the 56th Annual Meeting of the Institute of Navigation.

[8] Betz J.W., Shnidman N.R. Receiver processing losses with bandlimiting and one-bit sampling. Proceedings of the 20th International Technical Meeting of the Satellite Division of the Institute of Navigation (ION GNSS 2007).

[9] Hegarty C.J., Cerruti A.P. Results from an analytical model for GNSS receiver implementation losses. Proceedings of the 23rd International Technical Meeting of the Satellite Division of the Institute of Navigation (ION GNSS 2010).

[10] Betz J.W., Kolodziejski K.R. (2009). Generalized theory of code tracking with an early-late discriminator part I: Lower bound and coherent processing. IEEE Trans. Aerosp. Electron. Syst..

[11] Betz J.W., Kolodziejski K.R. (2009). Generalized theory of code tracking with an early-late discriminator part II: Noncoherent processing and numerical results. IEEE Trans. Aerosp. Electron. Syst..

[12] Lohan E.S. Analytical performance of CBOC-modulated Galileo E1 signal using sine BOC(1,1) receiver for mass-market applications. Proceedings of the Position Location and Navigation Symposium (PLANS), 2010 IEEE/ION.

[13] Anantharamu P.B., Borio O., Lachapelle G. (2011). Space-Time Equalization Techniques for New GNSS Signals. Ph.D Thesis.

[14] Yao Z., Lu M. (2011). Lower bound on spreading code tracking error under unmatched de-spreading mode. Electron. Lett..

[15] Proakis J., Salehi M. (2007). Digital Communications.

[16] Thayaparan S., Ng T.-S., Wang J. (2000). Half-sine and triangular despreading chip waveforms for coherent delay-locked tracking in DS/SS systems. IEEE Trans. Commun..

[17] Wu X., Ling C., Xiang H. Despreading chip waveform design for coherent delay-locked tracking in DS/SS systems. Proceedings of the IEEE International Conference on Communications, 2002, ICC 2002.

[18] Kaplan E., Hegarty C. (2005). Understanding GPS: Principles and Applications.

[19] James J.S., Penina A., Parkinson B.W. (1996). Per Enge “Multipath Effect”, Global Positioning System: Theory and Applications, Volume I; Progress in Astronautics and Aeronautics.

[20] Yao Z., Lu M. Constant Envelope Combination for Components on Different Carrier Frequencies with Unequal Power Allocation. Proceedings of the ION ITM 2013.

[21] Yao Z., Lu M. Design, Implementation, and Performance Analysis of ACE-BOC Modulation. Proceedings of the ION GNSS+ 2013.

[22] Yao Z., Zhang J., Lu M. (2016). ACE-BOC: Dual-frequency Constant Envelope Multiplexing for Satellite Navigation. IEEE Trans. Aerosp. Electron. Syst..

[23] Shivaramaiah N.C., Dempster A.G., Rizos C. Hybrid tracking loop architectures for the Galileo E5 signal. Proceedings of the European Navigation Conference on Global Navigation Satellite Systems ENC GNSS.

[24] Hein G.W., Avila-Rodriguez J.-A., Wallner S., Pratt A.R., Owen J., Issler J.-L., Betz J.W., Hegarty C.J., Lenahan L.S., Rushanan J.J. (2006). MBOC: The new optimized spreading modulation recommended for GALILEO L1 OS and GPS L1C. Proc. IEEEION PLANS.

[25] Rodríguez J.Á.Á., Hein G.W., Wallner S., Issler J.-L., Ries L., Lestarquit L., Latour A., de Godet J., Bastide F., Pratt T. (2008). The MBOC modulation: The final touch to the Galileo frequency and signal plan. Navigation.

[26] Yao Z., Lu M., Feng Z.M. (2010). Quadrature multiplexed BOC modulation for interoperable GNSS signals. Electron. Lett..

[27] Yao Z., Lu M. Optimized modulation for Compass B1-C signal with multiple processing modes. Proceedings of the ION GNSS 24th International Technical Meeting of the Satellite Division.

